# Endoplasmic Reticulum Stress/Ca^2+^-Calmodulin-Dependent Protein Kinase/Signal Transducer and Activator of Transcription 3 Pathway Plays a Role in the Regulation of Cellular Zinc Deficiency in Myocardial Ischemia/Reperfusion Injury

**DOI:** 10.3389/fphys.2021.736920

**Published:** 2022-01-05

**Authors:** Huanhuan Zhao, Dan Liu, Qiumei Yan, Xiyun Bian, Jing Yu, Jingjing Wang, Xinxin Cheng, Zhelong Xu

**Affiliations:** ^1^Department of Physiology and Pathophysiology, Tianjin Medical University, Tianjin, China; ^2^Central Laboratory, Tianjin, China; ^3^Tianjin Key Laboratory of Epigenetics for Organ Development in Preterm Infants, The Fifth Central Hospital of Tianjin, Tianjin, China

**Keywords:** zinc deficiency, ischemia/reperfusion (I/R) injury, ER stress, CaMKII, STAT3

## Abstract

Zinc homeostasis has been known to play a role in myocardial ischemia/reperfusion (I/R) injury, but the precise molecular mechanisms regulating the expression of ZIP transporters during reperfusion are still unclear. The aim of this study was to determine whether ER Stress/CaMKII/STAT3 pathway plays a role in the regulation of cellular zinc homeostasis. Zinc deficiency increased mRNA and protein expressions of the ER stress relevant markers Chop and Bip, and STAT3 phosphorylation in H9c2 or HL-1 cells, an effect that was abolished by ZnCl_2_. ER calcium concentration [(Ca^2+^)_ER_] was decreased and cytosolic calcium concentration [(Ca^2+^)_I_] was increased at the condition of normoxia or ischemia/reperfusion, indicating that zinc deficiency triggers ER stress and Ca^2+^ leak. Further studies showed that upregulation of STAT3 phosphorylation was reversed by Ca^2+^ chelator, indicating that intracellular Ca^2+^ is important for zinc deficiency-induced STAT3 activation. In support, zinc deficiency enhanced ryanodine receptors (RyR), a channel in the ER that mediate Ca^2+^ release, and Ca^2+^-calmodulin-dependent protein kinase (CaMKII) phosphorylation, implying that zinc deficiency provoked Ca^2+^ leak from ER via RyR and p-CaMKII is involved in STAT3 activation. Moreover, inhibition of STAT3 activation blocked zinc deficiency induced ZIP9 expression, and resulted in increased Zn^2+^ loss in cardiomyocytes, further confirming that STAT3 activation during reperfusion promotes the expression of ZIP9 zinc transporter to correct the imbalance in zinc homeostasis. In addition, suppressed STAT3 activation aggravated reperfusion injury. These data suggest that the ER Stress/CaMKII/STAT3 axis may be an endogenous protective mechanism, which increases the resistance of the heart to I/R.

## Introduction

Myocardial ischemia/reperfusion (I/R) injury is a very common cardiovascular disease with a high mortality rate (Reed et al., 2017). It has been reported that zinc loss upon reperfusion contributes to myocardial ischemia/reperfusion (I/R) injury (McIntosh et al., 2010). Zinc is a trace element, which participates in the metabolism of many enzymes and it is also the basis for regulating nucleic acid metabolism, protein synthesis and the structure and function of transcription factors (Berg and Shi, 1996). The imbalance of zinc homeostasis will lead to immune deficiency, growth inhibition, neurological dysfunction, and susceptibility to infectious diseases (Prasad et al., 1961; Tapazoglou et al., 1985; Vallee and Falchuk, 1993). Intracellular zinc homeostasis is closely controlled by two families of zinc transporters: ZnT (SLC30) and ZIP (SLC39). So far, 10 ZnT and 14 ZIP transporters have been identified, which have the opposite effect in zinc homeostasis (Liuzzi and Cousins, 2004). ZnT family transporters promote zinc outflow from cytoplasm into organelles or across the plasma membrane to reduce cytosolic free zinc levels, while ZIP transporters facilitate zinc inflow from the extracellular space or organelles into cytoplasm to increase cytosolic free zinc levels. Our previous studies demonstrated that ZIP2 expression was increased at reperfusion in *in vivo* mouse hearts, an effect that was abolished by ZnCl_2_, indicating that the increased expression of ZIP2 during reperfusion was caused by the loss of zinc. In addition, ZIP2 gene knockout significantly exacerbated myocardial I/R injury, while ZIP2 overexpression could reduce I/R injury, indicating that ZIP2 is cardioprotective against I/R injury by correcting zinc dyshomeostasis (Du et al., 2019). Obviously, ZIP transporters play a significant role in the maintenance of zinc homeostasis in cardiomyocytes during reperfusion. However, the precise molecular mechanisms regulating the expression of ZIP transporters during reperfusion is still unclear.

The nuclear transcriptional factor signal transducer and activator of transcription 3 (STAT3) is important for cellular proliferation, differentiation, and survival. STAT3 is activated by tyrosine phosphorylation (Tyr705), leading to dimerization and subsequent translocation into the nucleus to promote gene expression through the interaction with regulatory elements (Fu et al., 1992; Schindler et al., 1992; Ihle, 1995). It has been reported that STAT3 plays a part in epithelial-mesenchymal transition in zebra fish by targeting ZIP6 (Yamashita et al., 2004). Proinflammatory cytokines, such as IL1β and IL6 can stimulate STAT3 to up-regulate ZIP14 and ZIP6 and facilitate cellular zinc influx (Cousins et al., 2006). Our latest research shows that STAT3 regulates ZIP2 expression in I/R. However, how zinc deficiency acts on STAT3 activation in the myocardial ischemia/reperfusion injury remain unknown.

As an important intracellular calcium reservoir, endoplasmic reticulum (ER) plays an important role in multiple key cellular functions, including calcium homeostasis, the synthesis of major structural lipids, and the folding of membrane and secreted proteins (Brodsky, 2012). The accumulation of misfolded proteins can lead to a variety of effects, such as ER stress and the subsequent activation of the unfolded protein response (UPR) (Hetz, 2012). Zinc is essential for normal ER function, as zinc deficiency can cause ER stress (Homma et al., 2013; Nguyen et al., 2013). ER stress has been proposed to activate STAT3 (Ahyi et al., 2013; Meares et al., 2014). Studies have reported that IRE1α and PERK can promote STAT3 activation to increase the expression of anti-apoptotic proteins, thus promoting cell survival (Cubillos-Ruiz et al., 2017). So it is possible that ER stress/STAT3 plays a role in regulating zinc transporter expression in response to cellular zinc deficiency. Ca^2+^/CaM-dependent protein kinase II (CaMKII) is a serine/threonine-specific protein kinase that is regulated by the Ca^2+^/calmodulin complex. Studies have shown that reperfusion can initiate ER stress, leading to increase in intracellular Ca^2+^ release and the activation of the CaMKII (Timmins et al., 2009; Wang et al., 2016; Sheng et al., 2018). Accumulating studies reveals that there is cross-talk between STAT3 and CaMKII (Ma et al., 2017). For example, a recent study defined CaMKII as an upstream effector, which showed that CaMKII activation during stress leads to releasing STAT3, and allows for its translocation to nucleus to alter gene expression (Unudurthi et al., 2018). However, whether CaMKII/STAT3 is involved in the regulation of zinc homeostasis has not yet been reported.

The purpose of this study was to identify whether ER Stress/CaMKII/STAT3 pathway plays a role in the regulation of cellular zinc homeostasis. Here we reveal that zinc deficiency activates STAT3 by ER stress-induced Ca^2+^ release and subsequent CaMKII activation, leading to enhancement of the transcriptional activity of the ZIP family zinc transporter genes. These data suggest that ER Stress/CaMKII/STAT3 pathway responses to decrease in intracellular free zinc and promotes the expression of the ZIP genes that are required for zinc import leading to the protection against cellular zinc deficiency.

## Materials and Methods

### Chemicals

N,N,N′,N′ -Tetrakis (2-pyridylmethyl) ethylenediamine (TPEN) and thapsigargin (TG) were obtained from Sigma (St. Louis, MO, United States). BAPTA-AM, EGTA-AM, H89, 2APB, and stattic were purchased from MCE (NJ, United States). KN-93 and KN-92 were obtained from Selleck (Houston, TX, United States). Antibodies including anti-p-STAT3, -STAT3, -p-CaMKII, -CaMKII, -GAPDH, and the secondary antibody were obtained from Cell Signaling Technology (Beverly, MA, United States). Anti-IP3R,-p-RyR2 and -SERCA2 were purchased from Abcam (Cambridge, United Kingdom). Anti-RyR2 was purchased from Proteintech Group (Chicago, IL, United States). Anti-ZIP9 was obtained from Biorbyt (Cambridge, United Kingdom). Fluorescence dyes were obtained from Invitrogen (Carlsbad, CA, United States).

### Cell Culture

Rat heart tissue-derived H9c2 cardiac myoblast cell line and murine atrial tumor-derived HL-1 cardiomyocytes were purchased from ATCC. H9c2 cells were cultured and maintained with DMEM supplemented with 10% FBS and 100 U penicillin-streptomycin in a cell culture incubator at 37°C in a humidified 5% CO_2_-95% air atmosphere. HL-1 cells were grown at 37°C with 5% CO_2_ in claycomb medium (Sigma-Aldrich; Merck KGaA) containing 10% FBS (Gibco; Thermo Fisher Scientific, Inc.), 100 U/ml penicillin, 100 μg/ml streptomycin, 2 mM l-glutamine, and 0.1 mM norepinephrine.

### Animals

Male mice (C57BL/6J, 8–10 weeks old) were obtained from the Institute of Laboratory Animal Science, Chinese Academy of Medical Sciences (Beijing, China). All the treatments and subsequent analyses were conducted in a blind fashion and in accordance with the NIH Guide for the Care and Use of Laboratory Animals (Eighth Edition). The animal experiments have been approved by the Tianjin Medical University Animal Care and Use Committee.

### Hypoxia/Reoxygenation

To induce H/R injury, cells cultured in a 6-well plate filled with the hypoxia medium (1 g L^–1^ glucose without serum) were exposed to hypoxia (1% O_2_) by placing the plate in a humidified hypoxia glove box (Coy Laboratory Products Inc., Grass Lake, MI, United States) for 4 h. Then the normal DMEM (4.5 g L^–1^ glucose and 10% serum) replaced the hypoxia medium and cells were cultured in an incubator under normoxic conditions (room air with 5% CO_2_) for 2 h.

### *In vivo* Mouse Heart Ischemia/Reperfusion

Male mice (8–10 weeks) were anesthetized with sodium pentobarbital (80mg/kg, i.p.), intubated through atracheotomy, and aerated with positive end-expiratory pressure of 3 cm H_2_O. The adequacy of anesthesia was monitored with the corneal and withdrawal reflexes. The ventilation frequency was 110 breaths per minute, and the tidal volume was 135–150 μl. After left thoracotomy, the left anterior descending coronary artery (LAD) was surrounded by 7-0 Prolene line, and then passed through a small plastic tube. After that, tighten the tubing against the heart surface in order to induce ischemia. At this time, myocardial pallor and ST-segment elevation of electrocardiogram can be observed with naked eyes, which is confirmed as local ischemia. Mice hearts were occluded LAD for 30 min and then reperfused for 30 min. However, the sham operation group was also sutured under LAD but not occluded. At the end of experiments, the mice were anesthetized with sodium pentobarbital again, then euthanized with cervical dislocation, and the left ventricle below ligation point were immediately collected for Western blotting analysis.

### Measurement of Infarct Size

Myocardial infarct size was measured by Evans Blue and triphenyltetrazolium chloride (TTC) double staining methods. Mice hearts were occluded LAD for 30 min and then reperfused for 2 h. At the end reperfusion, Evans Blue was injected into the heart through the thoracic aorta. Hearts were excised and sliced. The slices were incubated in 1% TTC at 37°C for 20 min and fixed with 10% formalin at room temperature. Infarct size was measured with ImageJ in a single blind mode and was presented as a percentage of the risk zone.

### Western Blotting Analysis

Proteins were separated by SDS-polyacrylamide gel, and then transferred to a PVDF membrane. After blocking the non-specifc sites with non-fat milk, each membrane was incubated overnight at 4°C with a primary antibody (1:1,000). The membranes were washed and incubated for 90 min at room temperature with a secondary antibody (1:2,000). Protein bands were visualized by the ECL method.

### Real-Time Quantitative PCR

Total mRNA were isolated from each of experimental groups using Trizol reagent. Reverse transcription (RT) was performed to convert RNA into cDNA by using reverse transcriptase. Primers used to determine the expression of ERS related genes and ZIP9 were described in Supplementary Table 1. mRNA expressions were evaluated by quantitative RT-PCR using EvaGreen 2X qPCR MasterMix (abm, Richmond, CA, United States) and Bio-Rad CFX96-Touch Real-Time PCR Systems.

### Measurement of Ca^2+^ Concentrations

Endoplasmic reticulum and free Ca^2+^ concentrations in H9c2 or HL-1 cells and mice hearts were detected by Mag-fluo-4 (5 μM) and Fluo-4 (5 μM), respectively. After treatment, H9c2 or HL-1 cells were stained with Mag-fluo-4 (5 μM) and Fluo-4 (5 μM) at 37°C for 30 min. Mice hearts were extracted, frozen, and cut into 7-μm sections. Cardiomyocytes were also stained with the fluorescence probes at 37°C for 30 min and washed twice with PBS. Then the fluorescence was detected by FACSVERSE Flow Cytometer (BD Biosciences, CA, United States) or a laser scanning confocal microscope (Olympus, Tokyo, Japan). A 488-nm line of helium-neon laser line excited the green fluorescence, which imaged through a 525-nm-long path filter.

### Measurement of Zinc Concentrations in Cardiac Tissue

After 30 min of ischemia and then 30 min of reperfusion, the cardiac tissue was pre-dried, weighed, and completely nitrated by 2 mL 65% HNO_3_ at 120°C for 30 min. Then the samples were dissolved in 4 mL mineral-free water. The concentration of zinc was measured by using inductively coupled plasma optical emission spectroscopy (ICPOES, Optima™ 8000, Perkin Elmer, CT, United States) at a wave-length of 206.200 nm. The detection limit is about 1 ppb. Tissue concentrations were expressed as μg/g dry weight.

### Cytotoxicity Lactate Dehydrogenase Assay

H9c2 or HL-1 cells were seeded in 96-well plates (1 × 10^4^ cells/ml). After treatment, culture medium was collected and Lactate dehydrogenase (LDH) release was detected using the CK12 kit (DOJINDO) according to the manufacturer’s instruction. Absorbance was measured at a wave-length of 490nm.

### Measurements of Cardiac Marker Enzymes

Serum myocardial injury marker, cardiac troponin I (cTnI), creatine kinase-MB (CK-MB), and lactate dehydrogenase (LDH) were detected by ELISA (Bio-Swamp, China).

### Experimental Protocols

H89 (10 mg/kg), KN93 (10 mg/kg), BAPTA-AM (10 mg/kg), or Stattic (3 mg/kg) were injected 5 min before reperfusion and continued for 30 min or 2 h through the tail vein. H9c2 or HL-1 cells were subjected to 4 h of hypoxia followed by 2 h of reoxygenation or exposed to TPEN for 2 h. KN93, KN92, H89, BAPTA-AM, EGTA-AM, Stattic (10 μM) or 2APB (100 μM) were applied 2 h before exposure to TPEN for 2 h or 30 min before the onset of reoxygenation. ZnCl_2_ (10 μM) and ionophore pyrithione (4 μM) was applied 1 h after exposure to TPEN for 1 h. Cells were exposed to 50 nM thapsigargin (TG) for 4 h.

### Quantification and Statistical Analysis

Data are expressed as mean ± SEM and the number of experimental replications is indicated in the figure legends. Statistical difference was determined using Student *t*-test or ANOVA followed by Tukey’s test. Statistical significance was defined as *p* < 0.05. SigmaStat 3.5 was used for statistical analysis.

## Results

### Zinc Deficiency Can Induce Endoplasmic Reticulum Stress and Ca^2+^ Leak

**FIGURE 1:**
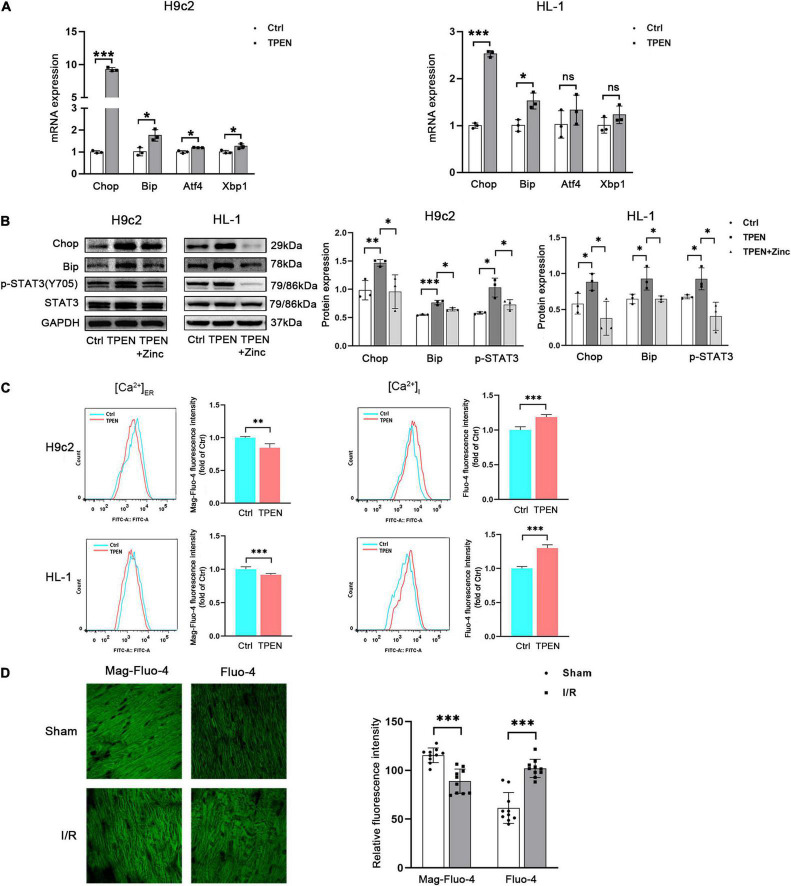
Zinc deficiency can induce ER Stress and Ca^2+^ leak. **(A)** Cells were exposed to TPEN (10 μM, *n* = 3) for 4 h. **(B)** TPEN was applied 1 h before exposure to ZnCl_2_ (10 μM, *n* = 3) and ionophore pyrithione (4 μM) for 1 h. **(C)** ER and free Ca^2+^ concentrations in cells were labeled with Mag-fluo4 (5 μM, *n* = 3) and Fluo-4 (5 μM, *n* = 3), respectively, and detected with confocal microscopy. **(D)** Mouse hearts were ischemic for 30 min and then reperfused for 30 min. Cardiomyocytes were labeled with Mag-fluo4 (5 μM) and Fluo-4 (5 μM), respectively (*n* = 10). *p < 0.05, ^**^p < 0.01, and ^***^p < 0.001. ns, no significant difference *P* > 0.05.

Our recent studies have shown that STAT3 is activated during reperfusion and protects the heart from I/R injury by up-regulating Zip2 protein expression (Du et al., 2019). In order to determine how zinc deficiency acts on STAT3 activation in the myocardial ischemia/reperfusion injury, RNA-seq analysis was performed. The pathway enrichment data suggest that zinc deficiency affects the protein processing in ER (Supplementary Figure 1). Zinc is required for normal ER function. To investigate whether zinc depletion can provoke ER stress, H9c2 or HL-1 cells were exposed to TPEN (a selective zinc chelator) for 4 h. TPEN increased mRNA expressions of the ER stress relevant markers CHOP and Bip in two cell lines (Figure 1A). TPEN has the highest affinity for Zn^2+^, it can also bind to other heavy metals such as iron (Jackson and Kodanko, 2010). To verify that ER stress was induced by the reduction of intracellular zinc levels, ZnCl_2_ (10 μM) and ionophore pyrithione (4 μM) were applied 1 h after the exposure to TPEN for 1 h. TPEN-induced CHOP, Bip protein expressions and STAT3 phosphorylation were inhibited by ZnCl_2_ (Figure 1B). Moreover, ER calcium concentration [(Ca^2+^)_ER_] was decreased and cytosolic calcium concentration [(Ca^2+^)_I_] was increased under the condition of normoxia (Figure 1C) or ischemia/reperfusion (Figure 1D), implying that the reduction of zinc levels in ER triggers ER stress and Ca^2+^ leak.

### Calcium Is Responsible for Zinc Deficiency-Induced Signal Transducer and Activator of Transcription 3 Activation

**FIGURE 2:**
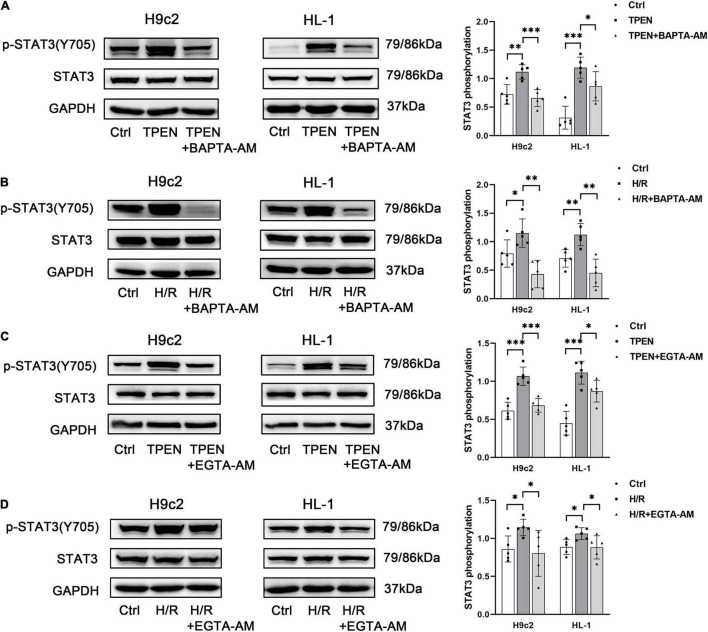
Calcium is responsible for zinc deficiency-induced STAT3 activation. BAPTA-AM (10 μM) **(A,B)** or EGTA-AM (10 μM) **(C,D)** was applied 2 h before exposure to TPEN for 2 h or 30 min before the onset of reoxygenation (*n* = 5). *p < 0.05, ^**^p < 0.01, and ^***^p < 0.001.

Abnormal Zn^2+^ homeostasis has been reported to be associated with dysregulation of intracellular Ca^2+^ release (Reilly-O’Donnell et al., 2017). Since zinc deficiency can trigger both Ca^2+^ leak and STAT3 activation, it is possible that Ca^2+^ is involved in zinc deficiency-induced STAT3 activation. To test this hypothesis, we determined the effect of BAPTA-AM, an intracellular Ca^2+^ chelator, on zinc deficiency-induced STAT3 activation. BAPTA-AM (10 μM) was applied 2 h before exposure to TPEN for 2 h or 30 min before the onset of reoxygenation. As shown in Figures 2A,B, BAPTA-AM reduced TPEN or H/R-induced STAT3 phosphorylation. To confirm this observation, EGTA-AM, a membrane permeable form of EGTA, was applied as above. EGTA-AM also reversed TPEN or H/R-induced STAT3 phosphorylation (Figures 2C,D), indicating that intracellular Ca^2+^ is responsible for zinc deficiency-induced STAT3 activation.

### Zinc Deficiency Activates Ca^2+^ Leak From Endoplasmic Reticulum via Ryanodine Receptor 2 and p-CaMKII Is Involved in Zinc Deficiency-Induced Signal Transducer and Activator of Transcription 3 Activation

**FIGURE 3:**
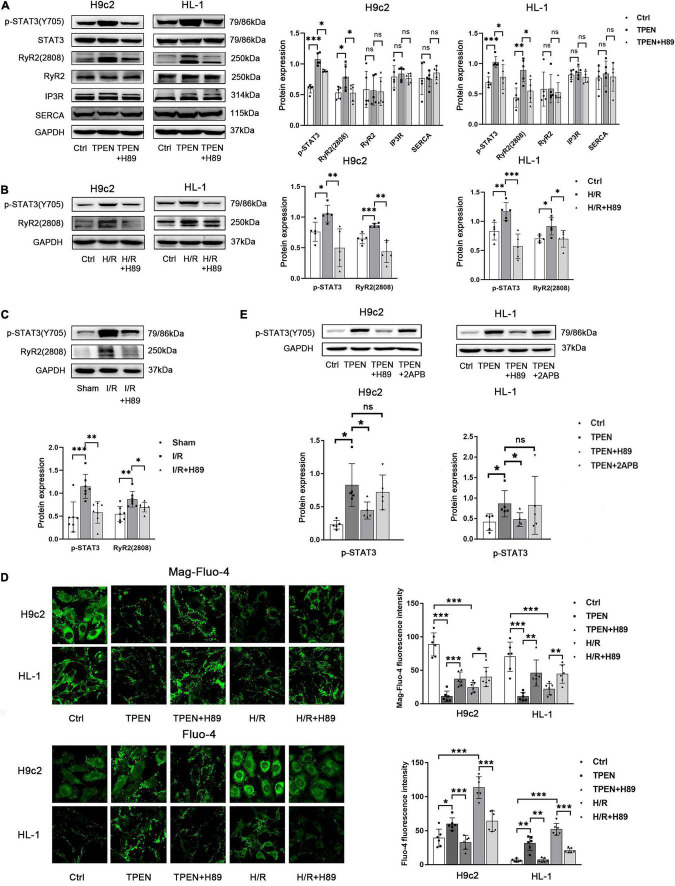
Zinc deficiency activates Ca^2+^ leak from ER via RyR2. **(A,B)** H89 (PKA inhibitor,10 μM, *n* = 5) was applied 2 h before exposure to TPEN for 2 h or 30 min before the onset of reoxygenation. **(C)** Mouse hearts were ischemic for 30 min and then reperfused for 30 min. H89 (10 mg/kg) was injected 5 min before reperfusion and continued for 30 min through the tail vein (*n* = 7). **(D)** H89 (10 μM, *n* = 6) was applied 2 h before exposure to TPEN for 2 h or 30 min before the onset of reoxygenation and labeled with Mag-fluo-4 (5 μM, *n* = 6) and Fluo-4 (5 μM, *n* = 6), respectively. **(E)** H89 (10 μM, *n* = 5) and 2APB (100 μM, *n* = 5) were applied 2 h before exposure to TPEN for 2 h. *p < 0.05, ^**^p < 0.01, and ^***^p < 0.001. ns, no significant difference *P* > 0.05.

The mechanism of zinc deficiency in regulating ER Ca^2+^ leak is poorly characterized. Ryanodine receptor (RyR) and inositol trisphosphate receptor (IP3R) are two channels in the ER that mediate Ca^2+^ release, while sarco(endo) plasmic reticulum Ca^2+^-ATPase (SERCA) is a pump that uptakes Ca^2+^ into SR(ER) (Popugaeva and Bezprozvanny, 2014; Zhai et al., 2020). Ca^2+^ release via the RyR and IP3R is facilitated by protein kinase A (PKA) (Leech et al., 2010). Then we examined how zinc deficiency shaped ER Ca^2+^ leak and further activated STAT3. H89 (PKA inhibitor, 10 μM) was applied 2 h before exposure to TPEN for 2 h or 30 min before the onset of reoxygenation. As shown in Figure 3A, the expression of IP3R and SERCA did not change in the setting of intracellular zinc depletion by TPEN, but the phosphorylation level of RyR2S2808 (PKA site) increased not only in the setting of intracellular zinc depletion by TPEN but also under hypoxia/reoxygen and ischemia/reperfusion conditions (Figures 3B,C). And H89 can inhibit TPEN, H/R or I/R-induced RyR2 and STAT3 phosphorylation. Moreover, H89 prevented the increase of cytoplasmic Ca^2+^ levels and the decrease of ER Ca^2+^ levels induced by TPEN or H/R (Figure 3D). Since PKA can also phosphorylate IP3R, 2-APB (a specific inhibitor of IP3 R, 10 μM) was applied. However, 2-APB treatment has no effect on TPEN induced STAT3 phosphorylation (Figure 3E). These results indicate that zinc deficiency can lead to RyR phosphorylation and increase its open probability, which result in an increased Ca^2+^ leakage from ER.

**FIGURE 4:**
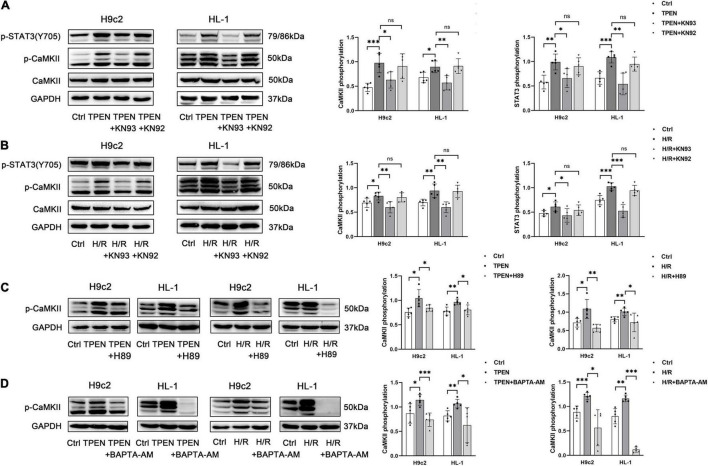
P-CaMKII is involved in zinc deficiency-induced STAT3 activation. KN93, KN92 **(A,B)**, H89 **(C),** or BAPTA-AM **(D)** (10 μM, *n* = 5) were applied 2 h before exposure to TPEN for 2 h or 30 min before the onset of reoxygenation. *p < 0.05, ^**^p < 0.01, and ^***^p < 0.001. ns, no significant difference *P* > 0.05.

CaMKII is a general integrator of Ca^2+^ signaling, which is activated when a large number of Ca^2+^ bind to calmodulin (CaM). To further determine whether CaMKII is involved in zinc deficiency-induced STAT3 activation, CaMKII inhibitor KN93 (10 uM) and its inactive analog KN92 (10 uM) were applied 2 h before exposure to TPEN for 2 h or 30 min before the onset of reoxygenation (Figures 4A,B). Indeed, inhibition of CaMKII with KN-93, but not the inactive analog KN92, decreased zinc deficiency-induced STAT3 phosphorylation, pointing that CaMKII activation is vital to STAT3 activation. In addition, H89 and BAPTA-AM also reversed TPEN or H/R-induced CaMKII phosphorylation, implying that CaMKII phosphorylation does require the release of Ca^2+^ to participate in the activation of downstream STAT3 (Figures 4C,D).

### Activated Signal Transducer and Activator of Transcription 3 Corrects the Imbalance of Zinc Homeostasis by Upregulating ZIP9 Expression

**FIGURE 5:**
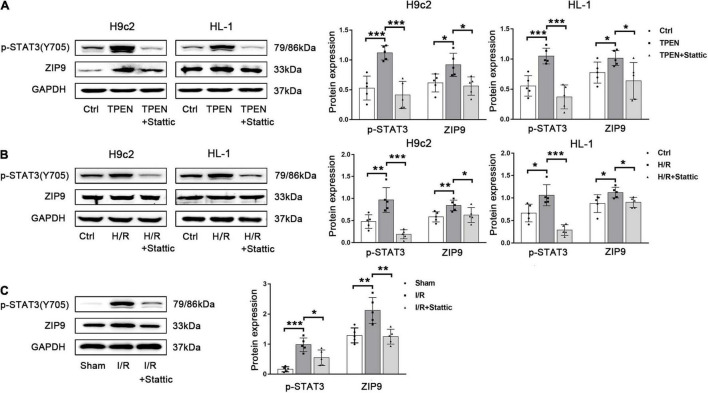
Zinc deficiency-induced STAT3 activation promotes ZIP9 expression. **(A,B)** Stattic (10 μM, *n* = 5) was applied 2 h before exposure to TPEN for 2 h or 30 min before the onset of reoxygenation. **(C)** Mouse hearts were ischemic for 30 min and then reperfused for 30 min. Stattic (3 mg/kg, *n* = 5) was injected 5 min before reperfusion and continued for 30 min through the tail vein. *p < 0.05, **p < 0.01, and ***p < 0.001.

Now more and more ZIP transporters have been found to play crucial roles in the maintenance of zinc homeostasis in cardiomyocytes during reperfusion. Our preliminary RNA sequencing data in H9c2 cells showed that ZIP9 expression is significantly increased after TPEN treatment (data not shown). Since STAT3 is activated upon zinc deficiency, it may play a role in the regulation of the Zip9 gene expression. Figures 5A,B showed that when STAT3 phosphorylation is inhibited by stattic, the expression of ZIP9 is also down-regulated. To test if cardiac zinc deficiency caused by ischemia/reperfusion can also provoke STAT3 activation and induce ZIP9 expression *in vivo*, mouse hearts were subjected to ischemia/reperfusion. Ischemia/reperfusion increased ZIP9 expression was reversed by stattic (Figure 5C), implying that zinc deficiency induced STAT3 activation leads to the upregulation of zinc importer ZIP9.

**FIGURE 6:**
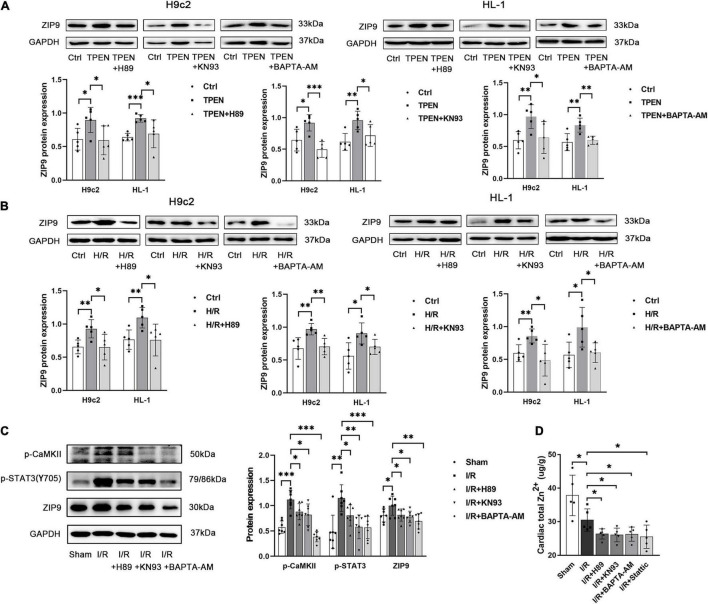
ER Stress/CaMKII is involved in the regulation of ZIP9 by STAT3 **(A,B)** H89, KN93, or BAPTA-AM (10 μM, *n* = 5) were applied 2 h before exposure to TPEN for 2 h or 30 min before the onset of reoxygenation. **(C)** Mouse hearts were ischemic for 30 min and then reperfused for 30 min. H89, KN93 or BAPTA-AM (10 mg/kg) were injected 5 min before reperfusion and continued for 30 min through the tail vein (*n* = 7). **(D)** Zn^2+^ levels were monitored with inductively coupled plasma optical emission spectroscopy (ICPOES, *n* = 5). *p < 0.05, ^**^p < 0.01, and ^***^p < 0.001.

To further assess whether ER Stress is involved in the regulation of ZIP9 by STAT3, ER stress inducer thapsigargin (TG) was applied. As shown in Supplementary Figure 2A, when TG induced strong ER stress, the mRNA expression of ZIP9 was also up-regulated. Besides, when STAT3 phosphorylation is inhibited by H89, KN93, BAPTA-AM, both the mRNA (Supplementary Figures 2B–D) and protein (Figures 6A–C) expression of ZIP9 are decreased, further confirming that ER Ca^2+^ leak and CaMKII activation act as upstream signals respond to zinc deficiency to activate STAT3 and promote zip9 expression.

In order to determine whether the ER stress/CaMKII/STAT3 pathway prevents myocardial Zn^2+^ loss, ICPOES was used to measure total Zn^2+^ levels in isolated rat hearts. Compared with the sham group, myocardial Zn^2+^ levels were significantly decreased after I/R. While the treatment of H89, KN93, BAPTA-AM, and Stattic resulted in increased Zn^2+^ loss in cardiomyocytes (Figure 6D). These results indicate the responding ER Stress/CaMKII/STAT3 pathway during reperfusion promotes the expression of ZIP9 zinc transporter to correct the imbalance in zinc homeostasis.

### Activated Signal Transducer and Activator of Transcription 3 Can Reduce Myocardial Reperfusion Injury

**FIGURE 7:**
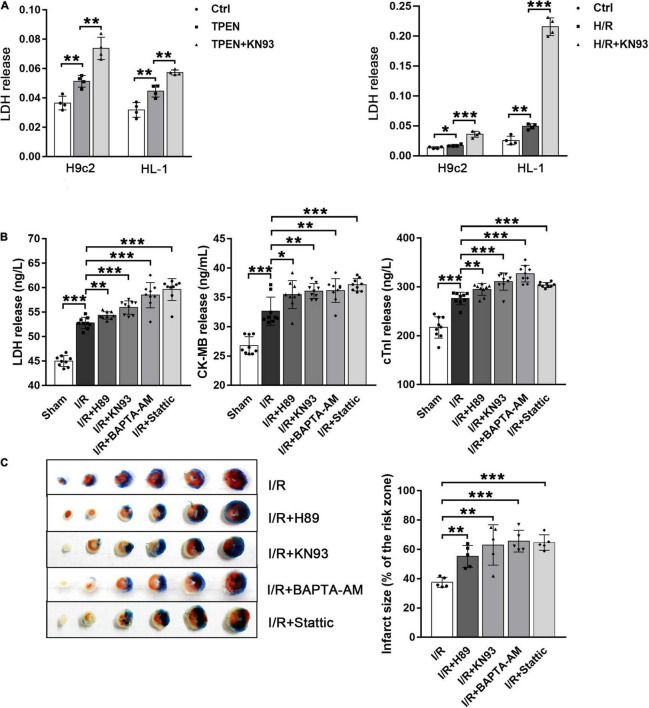
Activated STAT3 can reduce myocardial reperfusion injury. **(A)** KN93 (10 μM, *n* = 4) was applied 2 h before exposure to TPEN for 2 h or 30 min before the onset of reoxygenation. **(B)** Mouse hearts were ischemic for 30 min and then reperfused for 30 min. After I/R, the release of these myocardial injury markers were detected (*n* = 9). **(C)** Myocardial infarct size was assessed by TTC staining. Mouse hearts were subjected to 30 min ischemia followed by 2 h of reperfusion (*n* = 5). *p < 0.05, **p < 0.01, and ***p < 0.001.

To clarify the relationship between STAT3 activation and reperfusion injury, we first detected the release of the lactate dehydrogenase LDH in H9c2 and HL-1 cells. As shown in Figure 7A, TPEN or H/R can increase the release of LDH, while after KN93 treatment, the release of LDH further increased, indicating that inhibition of STAT3 activation can aggravate cell injury, since KN93 can reverse zinc deficiency induced STAT3 activation. Next, we confirmed this result in *in vivo* myocardial ischemia-reperfusion models. After I/R, the serum LDH, CK-MB, and cTnl activities, as well as infarct size, were increased, but after administration of H89, KN93, BATPA-AM and Stattic, cardiac injury were further increased (Figures 7B,C). These results suggest that ER Stress/CaMKII/STAT3 axis during myocardial ischemia-reperfusion can alleviate reperfusion injury, which may be an endogenous myocardial protective mechanism.

## Discussion

In this report, we demonstrate a new pathway response in the setting of intracellular zinc deficiency. Zinc deficiency activates STAT3 by ER stress-induced Ca^2+^ release and subsequent CaMKII activation, leading to enhancement of the transcriptional activity of the ZIP family zinc transporter genes. More importantly, the mechanism not only works in the setting of intracellular zinc depletion by TPEN but is also sensitive to zinc deficiency caused by hypoxia/reoxygen or ischemia/reperfusion.

Zinc has important structural, enzymatic, and regulatory functions (Ma et al., 2016). These functions of zinc require a tight control of zinc homeostasis. Zinc transporters strictly control intracellular zinc homeostasis in physiological conditions and zinc deficiency is related to many diseases (Hambidge and Krebs, 2007). Studies have demonstrated that ischemia/reperfusion in rat heart can lead to the decrease of intracellular zinc level, but supplementation of zinc can protect the heart, suggesting that the maintenance of zinc homeostasis is very important for the survival of heart during reperfusion (Karagulova et al., 2007). The cellular zinc homeostasis is maintained mainly by zinc transporters. Multiple ZIP family members, such as ZIP1, ZIP2, ZIP7, ZIP10, ZIP13, and ZIP14, were reported to response to zinc deficiency, with the mRNAs of these ZIPs accumulate when cells are exposed to zinc chelator TPEN (Cousins et al., 2003; Dietrich et al., 2017; Du et al., 2019; Thokala et al., 2019). Our preliminary RNA sequencing data in H9c2 cells showed that zinc deficiency can upregulate many ZIP transporters, ZIP9 is one of the most obvious ones. Many studies have reported that ZIP9 plays a critical role in regulating zinc homeostasis by transporting zinc across cell and organelle membranes into the cytoplasm (Matsuura et al., 2009; Taniguchi et al., 2013). In this study, we use ZIP9 as the representative of ZIP family to reduce work burden.

STAT proteins are part of the Janus tyrosine kinase (JAK)/STAT signal pathway (Darnell, 1997). Classical activation of STAT proteins occurs after cytokine bindings to cell surface receptors. The receptor dimerization induces activation of Janus tyrosine kinase (JAK) proteins and the activated JAK proteins activate STAT proteins through tyrosine phosphorylation (Tyr^705^for STAT3). Phosphorylated STAT proteins undergo dimerization and then STAT dimmers translocate into the nucleus and bind to specific DNA sequences of target genes to affect gene expression. STAT3 is an important protein in the signal transduction of cardioprotection. It has been reported that STAT3 activation protects the heart from I/R injury by reducing oxidative stress, inhibiting apoptosis, and inhibiting the inflammatory cascade (Wu et al., 2019, 2021; Yin et al., 2020). In addition to these results, we reveal that STAT3 activation can up-regulate the expression of ZIP family zinc transporters. In general, the ZIP family transporters transport zinc from cell exterior or intracellular organelles into the cytosol thereby increasing cytosolic free zinc. Therefore, STAT3 activation linking to ZIP transporter expression in the setting of cellular zinc deficiency is a cellular adaptive mechanism by which cells attempt to homeostatically compensate for zinc loss. In this study, STAT3 acts through its post translational phosphorylation at Tyr705 via ER stress-induced Ca^2+^ release and subsequent CaMKII activation (see below). This finding is very unique and provides a new STAT3 activation mechanism distinct from those previously proposed.

Zinc is required for ER function. Zinc deficiency can lead to an increase in the level of unfolded protein and ER stress because many proteins bind or acquire zinc in the ER (Ellis et al., 2004). Our preliminary RNA sequencing data suggest that zinc deficiency affects the protein processing in ER (Supplementary Figure 1). Further experimental data showed that zinc deficiency induced ER stress serves as an upstream signaling event leading to STAT3 activation and ZIP expression. ER Stress can activate STAT3 in several ways. A recent study has demonstrated that ER stress activated STAT3 in a PERK-dependent manner in glial cells (Meares et al., 2014). It has also been reported that ER transmembrane protein IRE1a can associate with STAT3 to enhancing STAT3 phosphorylation in mouse primary hepatocytes. (Liu et al., 2015). Besides, deletion of the ER resident thiol disulfide oxidoreductase ERp57 enhances STAT3 signaling (Coe et al., 2010). In this study, we reveal that ER stress induced Ca^2+^ release accounts for STAT3 activation.

Endoplasmic reticulum is an important intracellular calcium reservoir. The decrease of sarcoplasmic reticulum (SR) calcium content may help to protect myocardium from ischemia/reperfusion injury (Kumada et al., 1998). Ryanodine receptor (RyR) and inositol trisphosphate receptor (IP3R) in the ER can mediate Ca^2+^ release, and a sarco(endo) plasmic reticulum Ca^2+^-ATPase (SERCA) that uptakes Ca^2+^ into SR(ER) (Popugaeva and Bezprozvanny, 2014; Zhai et al., 2020). The signal network involved in the absorption and release of Ca^2+^ in the ER is very complex, and it has been suggested that protein kinase A (PKA) can regulate the uptake and release of Ca^2+^ in the ER by affecting IP3R, RyR and SERCA proteins, respectively (Leech et al., 2010). Sztretye et al. (2009) showed that zinc ion chelator TPEN could inhibit the increase of cytoplasmic calcium ion concentration by SERCA. Jung et al. (2007) showed that TPEN can activate the release of calcium ions by changing the activity of RyR. Lee et al. (2012) suggested that treatment of cells with IP3R inhibitor 2-APB could inhibit IL-31-induced STAT3 activation. In our study, we observed that the phosphorylation level of RyR2S2808 (PKA site), not the expression of IP3R, SERCA or total RyR, is increased in the setting of intracellular zinc depletion by TPEN. While PKA inhibitor H89 could reverse the up-regulation of p-RyR, the activation of CaMKII/STAT3 and the expression of downstream ZIP zinc transporter, indicating enhancing RyR phosphorylation is required for STAT3 activation. The different responses to TPEN can be attributed to various cell types, exposure time and/or threshold levels. TPEN is a specific cell-permeable heavy metal chelator. Although TPEN has the highest affinity for Zn^2+^, it can also chelate to other heavy metals (Jackson and Kodanko, 2010; Zhai et al., 2020). However, the enhanced phosphorylation of RyR may not be attributed to the chelation of other metal ions by TPEN, since p-RyR is also increased under pathophysiological hypoxia/reoxygen and ischemia/reperfusion conditions, and inhibition of RyR phosphorylation aggravates zinc ion loss during reperfusion (Karagulova et al., 2007; McIntosh et al., 2010). Obviously, the exact mechanism of zinc deficiency leading to RyR phosphorylation requires more researches.

It was shown that PKA-mediated phosphorylation of RyR2S2808 increases the Ca^2+^ binding affinity to RyR to enhance RyR opening probability (Houser, 2014). Increased ER Ca^2+^ leak activates CaMKII. In support, this study demonstrated that cytosolic Ca^2+^ chelated with BAPTA-AM significantly decreased CaMKII activation. CaMKII is composed of 4 isoforms (α, β, δ, and γ) which possesses expression patterns varying from tissues, mostly α and β in neurons, δ and some γ in cardiomyocytes (Gaertner et al., 2004; Kreusser et al., 2014; Hegyi et al., 2019). CaMKII activity is sustained by autophosphorylation at Thr286 (CaMK2α) or Thr-287 (β, δ, and γ) (Uniprot database). In this study, surprisingly, we detected a very significant increase in CaMKII phosphorylation at Thr286. Meanwhile, inhibition of CaMKII with KN-93, but not the inactive analog KN92, decreased STAT3 phosphorylation, pointing that CaMKII activation at Thr286 is vital to STAT3 activation. Contrary to our results, a large number of studies have shown that inhibiting CaMKII can reduce myocardial ischemia/reperfusion injury (Hong et al., 2010; Ling et al., 2013). Indeed, most groups detect phosphorylation at Thr287 CaMK2δ or CaMK2γ. It is possible that CaMK2α is the dominant isoform which autophosphorylation at Thr-286 that protects the heart from ischemia reperfusion injury. A lot more work are needed to study the mechanism of CaMKII in regulating STAT3.

In this study we have also demonstrated that ZIP9 is upregulated upon hypoxia/reoxygenation or ischemia-reperfusion in the heart or H9c2/HL-1 cells. One of our recent study has shown that ZIP2 was upregulated at reperfusion and protects the heart against ischemia-reperfusion injury (Du et al., 2019). Therefore, it is intriguing to explore the roles of ZIP9 in the setting of myocardial ischemia/reperfusion injury in future studies.

**FIGURE 8:**
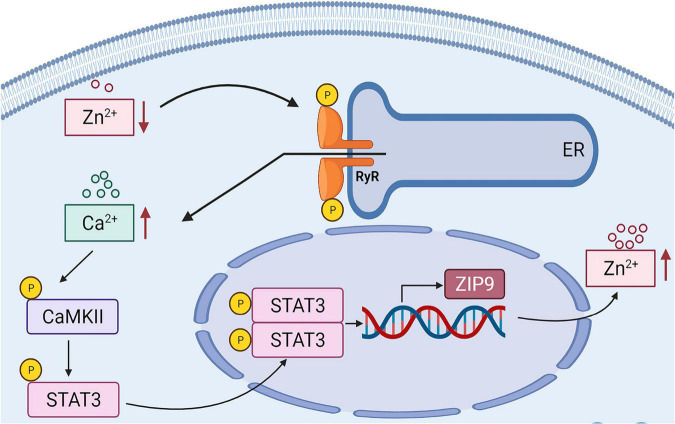
Cellular endogenous protective mechanism induced by zinc deficiency.

In conclusion (Figure 8), in this study, we demonstrated that zinc deficiency induced Ca^2+^ release can activate CaMKII at Thr286 which in turn leads to STAT3 activation. This is a new pathway which responses cellular zinc deficiency and promotes the transcription of the genes encoding for the zinc importers to compensate cellular zinc loss. The ER Stress/CaMKII/STAT3 axis provides new therapeutic targets for the treatment of diseases caused by zinc deficiency.

## Data Availability Statement

The original contributions presented in the study are included in the article/Supplementary Material, further inquiries can be directed to the corresponding authors.

## Ethics Statement

The animal study was reviewed and approved by Tianjin Medical University Animal Care and Use Committee.

## Author Contributions

HZ, DL, and XB performed the experiments with cells. QY, JW, and JY conducted animal experiments. XC participated in data discussion and manuscript preparation. ZX designed and supervised the study. All authors approved the final version of the manuscript for publication.

## Conflict of Interest

The authors declare that the research was conducted in the absence of any commercial or financial relationships that could be construed as a potential conflict of interest.

## Publisher’s Note

All claims expressed in this article are solely those of the authors and do not necessarily represent those of their affiliated organizations, or those of the publisher, the editors and the reviewers. Any product that may be evaluated in this article, or claim that may be made by its manufacturer, is not guaranteed or endorsed by the publisher.

## References

[B1] Ahyi A. N. Quinton L. J. Jones M. R. Ferrari J. D. Pepper-Cunningham Z. A. Mella J. R. (2013). Roles of STAT3 in protein secretion pathways during the acute-phase response. *Infect. Immun.* 81 1644–1653. 10.1128/iai.01332-12 23460517 PMC3647986

[B2] Berg J. M. Shi Y. (1996). The galvanization of biology: a growing appreciation for the roles of zinc. *Science* 271 1081–1085. 10.1126/science.271.5252.1081 8599083

[B3] Brodsky J. L. (2012). Cleaning up: ER-associated degradation to the rescue. *Cell* 151 1163–1167. 10.1016/j.cell.2012.11.012 23217703 PMC3521611

[B4] Coe H. Jung J. Groenendyk J. Prins D. Michalak M. (2010). ERp57 modulates STAT3 signaling from the lumen of the endoplasmic reticulum. *J. Biol. Chem.* 285 6725–6738. 10.1074/jbc.M109.054015 20022947 PMC2825467

[B5] Cousins R. J. Blanchard R. K. Popp M. P. Liu L. Cao J. Moore J. B. (2003). A global view of the selectivity of zinc deprivation and excess on genes expressed in human THP-1 mononuclear cells. *Proc. Natl. Acad. Sci. U.S.A.* 100 6952–6957. 10.1073/pnas.0732111100 12756304 PMC165811

[B6] Cousins R. J. Liuzzi J. P. Lichten L. A. (2006). Mammalian zinc transport, trafficking, and signals. *J. Biol. Chem.* 281 24085–24089. 10.1074/jbc.R600011200 16793761

[B7] Cubillos-Ruiz J. R. Bettigole S. E. Glimcher L. H. (2017). Tumorigenic and immunosuppressive effects of endoplasmic reticulum stress in cancer. *Cell* 168 692–706. 10.1016/j.cell.2016.12.004 28187289 PMC5333759

[B8] Darnell J. E. Jr. (1997). STATs and gene regulation. *Science* 277 1630–1635. 10.1126/science.277.5332.1630 9287210

[B9] Dietrich N. Schneider D. L. Kornfeld K. (2017). A pathway for low zinc homeostasis that is conserved in animals and acts in parallel to the pathway for high zinc homeostasis. *Nucleic Acids Res.* 45 11658–11672. 10.1093/nar/gkx762 28977437 PMC5714235

[B10] Du L. Zhang H. Zhao H. Cheng X. Qin J. Teng T. (2019). The critical role of the zinc transporter Zip2 (SLC39A2) in ischemia/reperfusion injury in mouse hearts. *J. Mol. Cell Cardiol.* 132 136–145. 10.1016/j.yjmcc.2019.05.011 31095941

[B11] Ellis C. D. Wang F. MacDiarmid C. W. Clark S. Lyons T. Eide D. J. (2004). Zinc and the Msc2 zinc transporter protein are required for endoplasmic reticulum function. *J. Cell Biol.* 166 325–335. 10.1083/jcb.200401157 15277543 PMC2172251

[B12] Fu X. Y. Schindler C. Improta T. Aebersold R. Darnell J. E. Jr. (1992). The proteins of ISGF-3, the interferon alpha-induced transcriptional activator, define a gene family involved in signal transduction. *Proc. Natl. Acad. Sci. U.S.A.* 89 7840–7843. 10.1073/pnas.89.16.7840 1502204 PMC49807

[B13] Gaertner T. R. Kolodziej S. J. Wang D. Kobayashi R. Koomen J. M. Stoops J. K. (2004). Comparative analyses of the three-dimensional structures and enzymatic properties of alpha, beta, gamma and delta isoforms of Ca2+-calmodulin-dependent protein kinase II. *J. Biol. Chem.* 279 12484–12494. 10.1074/jbc.M313597200 14722083

[B14] Hambidge K. M. Krebs N. F. (2007). Zinc deficiency: a special challenge. *J. Nutr.* 137 1101–1105. 10.1093/jn/137.4.1101 17374687

[B15] Hegyi B. Bers D. M. Bossuyt J. (2019). CaMKII signaling in heart diseases: emerging role in diabetic cardiomyopathy. *J. Mol. Cell Cardiol.* 127 246–259. 10.1016/j.yjmcc.2019.01.001 30633874

[B16] Hetz C. (2012). The unfolded protein response: controlling cell fate decisions under ER stress and beyond. *Nat. Rev. Mol. Cell Biol.* 13 89–102. 10.1038/nrm3270 22251901

[B17] Homma K. Fujisawa T. Tsuburaya N. Yamaguchi N. Kadowaki H. Takeda K. (2013). SOD1 as a molecular switch for initiating the homeostatic ER stress response under zinc deficiency. *Mol. Cell* 52 75–86. 10.1016/j.molcel.2013.08.038 24076220

[B18] Hong Y. Downey T. Eu K. W. Koh P. K. Cheah P. Y. (2010). A ‘metastasis-prone’ signature for early-stage mismatch-repair proficient sporadic colorectal cancer patients and its implications for possible therapeutics. *Clin. Exp. Metastasis* 27 83–90. 10.1007/s10585-010-9305-4 20143136

[B19] Houser S. R. (2014). Role of RyR2 phosphorylation in heart failure and arrhythmias: protein kinase A-mediated hyperphosphorylation of the ryanodine receptor at serine 2808 does not alter cardiac contractility or cause heart failure and arrhythmias. *Circ Res* 114 1320–1327; discussion1327. 10.1161/CIRCRESAHA.114.300569 24723657 PMC4040460

[B20] Ihle J. N. (1995). Cytokine receptor signalling. *Nature* 377 591–594. 10.1038/377591a0 7566171

[B21] Jackson C. S. Kodanko J. J. (2010). Iron-binding and mobilization from ferritin by polypyridyl ligands. *Metallomics* 2 407–411. 10.1039/c003414b 21072387

[B22] Jung C. Zima A. V. Szentesi P. Jona I. Blatter L. A. Niggli E. (2007). Ca2+ release from the sarcoplasmic reticulum activated by the low affinity Ca2+ chelator TPEN in ventricular myocytes. *Cell Calcium* 41 187–194. 10.1016/j.ceca.2006.06.009 16920191

[B23] Karagulova G. Yue Y. Moreyra A. Boutjdir M. Korichneva I. (2007). Protective role of intracellular zinc in myocardial ischemia/reperfusion is associated with preservation of protein kinase C isoforms. *J. Pharmacol. Exp. Ther.* 321 517–525. 10.1124/jpet.107.119644 17322024

[B24] Kreusser M. M. Lehmann L. H. Keranov S. Hoting M. O. Oehl U. Kohlhaas M. (2014). Cardiac CaM Kinase II genes delta and gamma contribute to adverse remodeling but redundantly inhibit calcineurin-induced myocardial hypertrophy. *Circulation* 130 1262–1273. 10.1161/CIRCULATIONAHA.114.006185 25124496 PMC4316667

[B25] Kumada Y. Yamamoto F. Yamamoto H. Ishikawa T. Kagisaki K. Hirose H. (1998). [Decreasing sarcoplasmic reticular calcium gives rise to myocardial protection–the effect of thapsigargin for myocardial protection under conditions of normothermia]. *Jpn. J. Thorac. Cardiovasc. Surg.* 46 368–374. 10.1007/BF03217757 9619037

[B26] Lee C. H. Hong C. H. Yu W. T. Chuang H. Y. Huang S. K. Chen G. S. (2012). Mechanistic correlations between two itch biomarkers, cytokine interleukin-31 and neuropeptide beta-endorphin, via STAT3/calcium axis in atopic dermatitis. *Br. J. Dermatol.* 167 794–803. 10.1111/j.1365-2133.2012.11047.x 22578170 PMC3482403

[B27] Leech C. A. Chepurny O. G. Holz G. G. (2010). Epac2-dependent rap1 activation and the control of islet insulin secretion by glucagon-like peptide-1. *Vitam. Horm.* 84 279–302. 10.1016/B978-0-12-381517-0.00010-2 21094904 PMC3009389

[B28] Ling H. Gray C. B. Zambon A. C. Grimm M. Gu Y. Dalton N. (2013). Ca2+/Calmodulin-dependent protein kinase II delta mediates myocardial ischemia/reperfusion injury through nuclear factor-kappaB. *Circ. Res.* 112 935–944. 10.1161/CIRCRESAHA.112.276915 23388157 PMC3673710

[B29] Liu Y. Shao M. Wu Y. Yan C. Jiang S. Liu J. (2015). Role for the endoplasmic reticulum stress sensor IRE1alpha in liver regenerative responses. *J. Hepatol.* 62 590–598. 10.1016/j.jhep.2014.10.022 25457211

[B30] Liuzzi J. P. Cousins R. J. (2004). Mammalian zinc transporters. *Annu. Rev. Nutr.* 24 151–172. 10.1146/annurev.nutr.24.012003.132402 15189117

[B31] Ma J. Zhao N. Zhu D. (2016). Bioabsorbable zinc ion induced biphasic cellular responses in vascular smooth muscle cells. *Sci. Rep.* 6:26661. 10.1038/srep26661 27248371 PMC4888653

[B32] Ma X. Meng Z. Jin L. Xiao Z. Wang X. Tsark W. M. (2017). CAMK2gamma in intestinal epithelial cells modulates colitis-associated colorectal carcinogenesis via enhancing STAT3 activation. *Oncogene* 36 4060–4071. 10.1038/onc.2017.16 28319059 PMC5509478

[B33] Matsuura W. Yamazaki T. Yamaguchi-Iwai Y. Masuda S. Nagao M. Andrews G. K. (2009). SLC39A9 (ZIP9) regulates zinc homeostasis in the secretory pathway: characterization of the ZIP subfamily I protein in vertebrate cells. *Biosci. Biotechnol. Biochem.* 73 1142–1148. 10.1271/bbb.80910 19420709

[B34] McIntosh R. Lee S. Ghio A. J. Xi J. Zhu M. Shen X. (2010). The critical role of intracellular zinc in adenosine A(2) receptor activation induced cardioprotection against reperfusion injury. *J. Mol. Cell Cardiol.* 49 41–47. 10.1016/j.yjmcc.2010.02.001 20144616 PMC2883651

[B35] Meares G. P. Liu Y. Rajbhandari R. Qin H. Nozell S. E. Mobley J. A. (2014). PERK-dependent activation of JAK1 and STAT3 contributes to endoplasmic reticulum stress-induced inflammation. *Mol. Cell Biol.* 34 3911–3925. 10.1128/mcb.00980-14 25113558 PMC4187715

[B36] Nguyen T. S. Kohno K. Kimata Y. (2013). Zinc depletion activates the endoplasmic reticulum-stress sensor Ire1 via pleiotropic mechanisms. *Biosci. Biotechnol. Biochem.* 77 1337–1339. 10.1271/bbb.130130 23748779

[B37] Popugaeva E. Bezprozvanny I. (2014). Can the calcium hypothesis explain synaptic loss in Alzheimer’s disease? *Neurodegen. Dis.* 13 139–141. 10.1159/000354778 24080896 PMC3946370

[B38] Prasad A. S. Halsted J. A. Nadimi M. (1961). Syndrome of iron deficiency anemia, hepatosplenomegaly, hypogonadism, dwarfism and geophagia. *Am. J. Med.* 31 532–546. 10.1016/0002-9343(61)90137-1 14488490

[B39] Reed G. W. Rossi J. E. Cannon C. P. (2017). Acute myocardial infarction. *Lancet* 389 197–210. 10.1016/s0140-6736(16)30677-8 27502078

[B40] Reilly-O’Donnell B. Robertson G. B. Karumbi A. McIntyre C. Bal W. Nishi M. (2017). Dysregulated Zn(2+) homeostasis impairs cardiac type-2 ryanodine receptor and mitsugumin 23 functions, leading to sarcoplasmic reticulum Ca(2+) leakage. *J. Biol. Chem.* 292 13361–13373. 10.1074/jbc.M117.781708 28630041 PMC5555195

[B41] Schindler C. Shuai K. Prezioso V. R. Darnell J. E. Jr. (1992). Interferon-dependent tyrosine phosphorylation of a latent cytoplasmic transcription factor. *Science* 257 809–813. 10.1126/science.1496401 1496401

[B42] Sheng M. Zhang G. Wang J. Yang Q. Zhao H. Cheng X. (2018). Remifentanil induces cardio protection against ischemia/reperfusion injury by inhibiting endoplasmic reticulum stress through the maintenance of zinc homeostasis. *Anesth. Analg.* 127 267–276. 10.1213/ANE.0000000000003414 29771714

[B43] Sztretye M. Almassy J. Deli T. Szentesi P. Jung C. Dienes B. (2009). Altered sarcoplasmic reticulum calcium transport in the presence of the heavy metal chelator TPEN. *Cell Calcium* 46 347–355. 10.1016/j.ceca.2009.10.002 19900703

[B44] Taniguchi M. Fukunaka A. Hagihara M. Watanabe K. Kamino S. Kambe T. (2013). Essential role of the zinc transporter ZIP9/SLC39A9 in regulating the activations of Akt and Erk in B-cell receptor signaling pathway in DT40 cells. *PLoS One* 8:e58022. 10.1371/journal.pone.0058022 23505453 PMC3591455

[B45] Tapazoglou E. Prasad A. S. Hill G. Brewer G. J. Kaplan J. (1985). Decreased natural killer cell activity in patients with zinc deficiency with sickle cell disease. *J. Lab. Clin. Med.* 105 19–22. 3968462

[B46] Thokala S. Bodiga V. L. Kudle M. R. Bodiga S. (2019). Comparative response of cardiomyocyte ZIPs and ZnTs to extracellular zinc and TPEN. *Biol. Trace Elem. Res.* 192 297–307. 10.1007/s12011-019-01671-0 30778755

[B47] Timmins J. M. Ozcan L. Seimon T. A. Li G. Malagelada C. Backs J. (2009). Calcium/calmodulin-dependent protein kinase II links ER stress with Fas and mitochondrial apoptosis pathways. *J. Clin. Invest.* 119 2925–2941. 10.1172/JCI38857 19741297 PMC2752072

[B48] Unudurthi S. D. Nassal D. Greer-Short A. Patel N. Howard T. Xu X. (2018). betaIV-Spectrin regulates STAT3 targeting to tune cardiac response to pressure overload. *J. Clin. Invest.* 128 5561–5572. 10.1172/JCI99245 30226828 PMC6264732

[B49] Vallee B. L. Falchuk K. H. (1993). The biochemical basis of zinc physiology. *Physiol. Rev.* 73 79–118. 10.1152/physrev.1993.73.1.79 8419966

[B50] Wang G. Huang H. Zheng H. He Y. Zhang Y. Xu Z. (2016). Zn(2+) and mPTP mediate endoplasmic reticulum stress inhibition-induced cardioprotection against myocardial ischemia/reperfusion injury. *Biol. Trace Elem. Res.* 174 189–197. 10.1007/s12011-016-0707-2 27106542

[B51] Wu J. W. Hu H. Li D. Ma L. K. (2021). Hypoxia-inducible factor 2-alpha-dependent induction of IL-6 protects the heart from ischemia/reperfusion injury. *Aging (Albany NY)* 13 3443–3458. 10.18632/aging.202276 33428604 PMC7906200

[B52] Wu L. Tan J. L. Chen Z. Y. Huang G. (2019). Cardioprotection of post-ischemic moderate ROS against ischemia/reperfusion via STAT3-induced the inhibition of MCU opening. *Basic Res. Cardiol.* 114:39. 10.1007/s00395-019-0747-9 31463567

[B53] Yamashita S. Miyagi C. Fukada T. Kagara N. Che Y. S. Hirano T. (2004). Zinc transporter LIVI controls epithelial-mesenchymal transition in zebrafish gastrula organizer. *Nature* 429 298–302. 10.1038/nature02545 15129296

[B54] Yin Q. Zhao B. Zhu J. Fei Y. Shen W. Liang B. (2020). JLX001 improves myocardial ischemia-reperfusion injury by activating Jak2-Stat3 pathway. *Life Sci.* 257:118083. 10.1016/j.lfs.2020.118083 32673665

[B55] Zhai X. Sterea A. M. Hiani Y. E. (2020). Lessons from the endoplasmic reticulum Ca(2+) Transporters-A cancer connection. *Cells* 9:1536. 10.3390/cells9061536 32599788 PMC7349521

