# Supplementary material for: Endoplasmic Reticulum Stress/Ca2+-Calmodulin-Dependent Protein Kinase/Signal Transducer and Activator of Transcription 3 Pathway Plays a Role in the Regulation of Cellular Zinc Deficiency in Myocardial Ischemia/Reperfusion Injury
Source: Front Physiol. 2022 Jan 5;12:736920. doi: 10.3389/fphys.2021.736920 (PMC8766834; doi:10.3389/fphys.2021.736920)
Supplement: Supplementary file 1 [file Data_Sheet_1.docx]

Supplementary Material


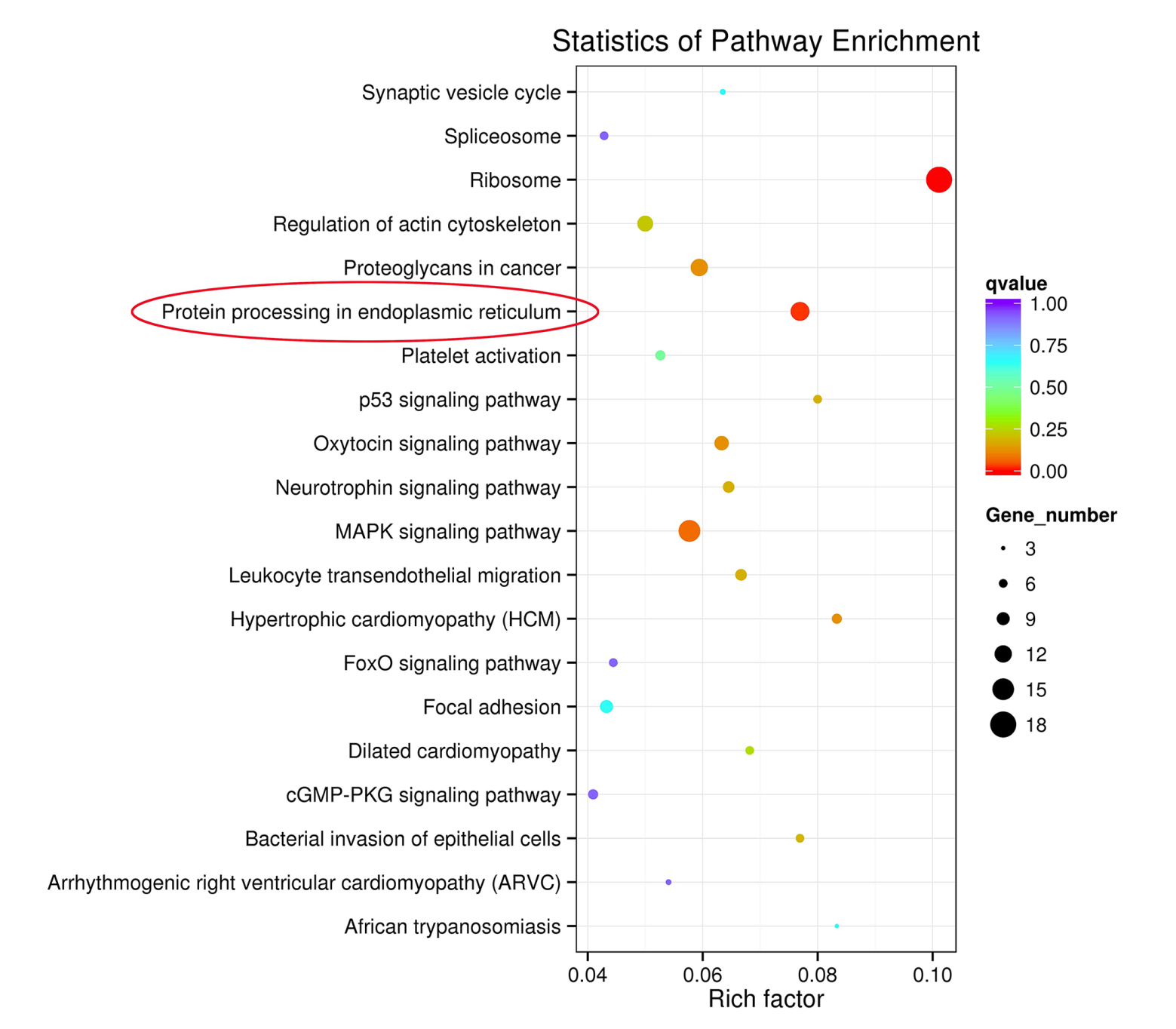


**Supplementary Figure S1.** The pathway enrichment data. H9c2 cells were exposed to TPEN (10 µM, n=3) for 4 h.


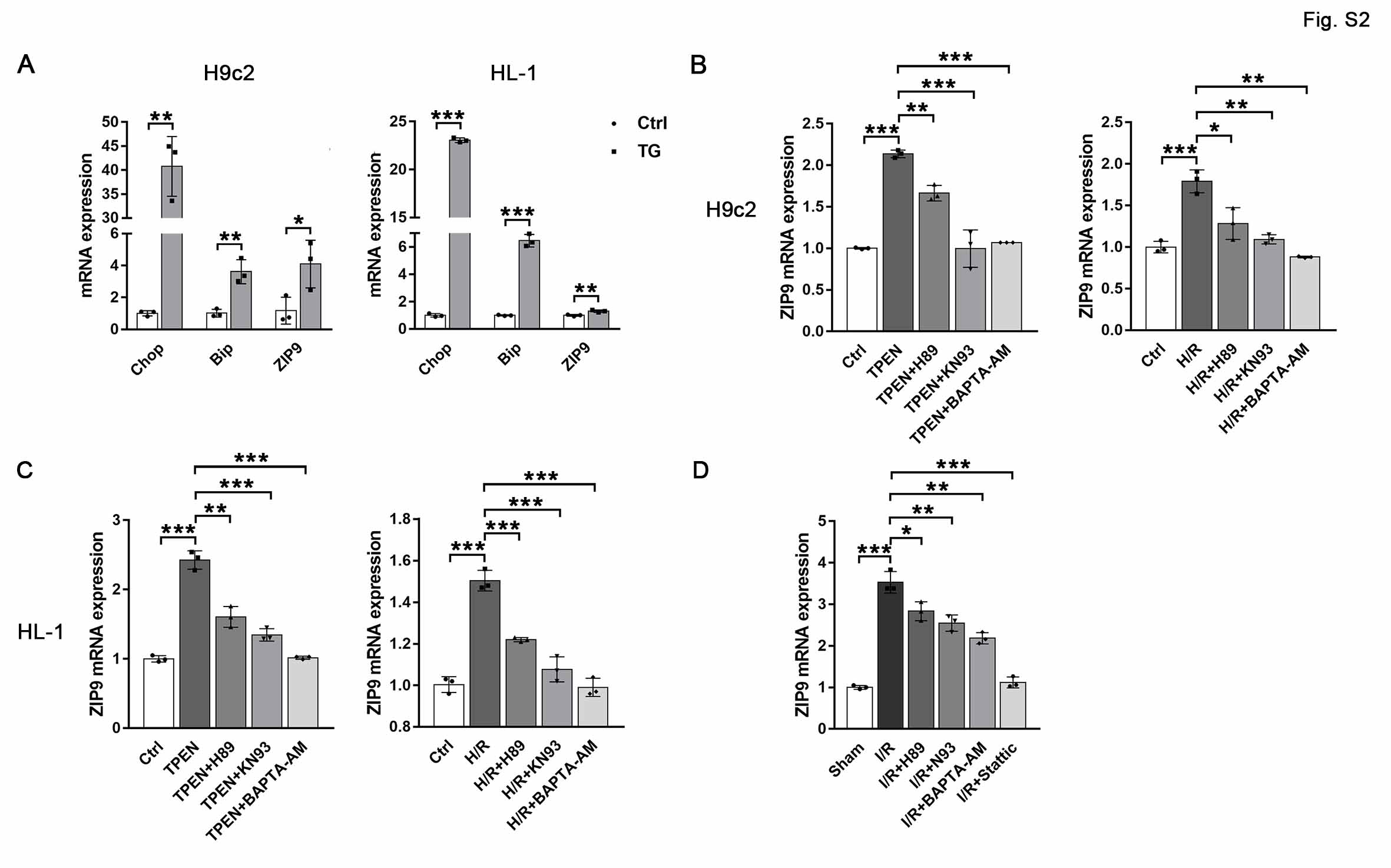


**Supplementary Figure S2.** Zinc deficiency-induced STAT3 activation promotes ZIP9 mRNA expression. **(A)** Cells were exposed to 50 nM thapsigargin (TG) for 4 h. **(B,C)** H89, KN93 or BAPTA-AM (10 μM, n=3) were applied 2 h before exposure to TPEN for 2 h or 30 min before the onset of reoxygenation. **(D)** Mouse hearts were ischemic for 30 minutes and then reperfused for 30 minutes. H89, KN93, BAPTA-AM (10 mg/kg, n=3) or Stattic (3 mg/kg, n=3) were injected 5 minutes before reperfusion and continued for 30 minutes through the tail vein.*p< 0.05, **p< 0.01, ***p< 0.01.

| **Genes** | **Forward primer** | **Reverse primer** |
| --- | --- | --- |
| **rChop** | CACACCTGAAAGCAGAAACC | GACCTCCTGCAGATCCTCAT |
| **rBip** | ATCATCAATGAGCCAACAGC | TTAGTGGCCACCACTTCAAA |
| **rXbp1** | GGATTTGGAAGAAGAGAACCA | TTCAGTAACCAGGGCATTCA |
| **rAtf4** | AAACCTCATGGGTTCTCCAG | GGTTTCCAGGTCATCCATTC |
| **mZip9** | TCTCAGAGGAGCGGCTGAAG | CTCCTTCCGGGACGATGACC |
| **mChop** | ATCCCAAAGCCCTCGCTCTC | TTTCCGCTCGTTCTCCTGCT |
| **mBip** | GCCGAGGAGGAGGACAAGAA | CCGACGCAGGAATAGGTGGT |
| **mAtf4** | GCCTGACTCTGCTGCTTACA | AGAGCCCAGGTAGGACTCTG |
| **mXbp1** | AACACGCTTGGGAATGGACA | CACCTGCTGCGGACTCA |
| **GAPDH** | GGTGATGCTGGTGCTGAGTA | ACTGTGGTCATGAGCCCTTC |

**Supplementary Table S1.** List of primers
